# Characterization of the Pig Gut Microbiome and Antibiotic Resistome in Industrialized Feedlots in China

**DOI:** 10.1128/mSystems.00206-19

**Published:** 2019-12-17

**Authors:** Chunlai Wang, Peng Li, Qiulong Yan, Liping Chen, Tiantian Li, Wanjiang Zhang, He Li, Changming Chen, Xiuyan Han, Siyi Zhang, Miao Xu, Bo Li, Xiaoxuan Zhang, Hongbo Ni, Yufang Ma, Bo Dong, Shenghui Li, Siguo Liu

**Affiliations:** a State Key Laboratory of Veterinary Biotechnology, Division of Bacterial Diseases, Harbin Veterinary Research Institute, Chinese Academy of Agricultural Sciences, Harbin, China; b Shenzhen Puensum Genetech Institute, Shenzhen, China; c Department of Microbiology, College of Basic Medical Sciences, Dalian Medical University, Dalian, China; d College of Animal Science and Veterinary Medicine, Heilongjiang Bayi Agricultural University, Daqing, China; University of California, San Diego

**Keywords:** industrialized feedlot, gene catalogue, pig gut microbiome, antibiotic resistance gene, antibiotic resistome

## Abstract

To characterize the diversity and richness and explore the function and structure of swine gut microbiome and resistome in common pig-farming feedlots, we sampled and metagenomic sequenced the feces of pigs from four different industrialized feedlots located in four distant provinces across China. Surprisingly, more than half of the nonredundant genes (1,937,648, 54.3%) in the current catalogue were newly found compared with the previously published reference gene catalogue (RGC) of the pig gut microbiome. Additionally, 16 high-completeness draft genomes were obtained by analyzing the dominant species on each feedlot. Notably, seven of these species often appeared in the human body sites. Despite a smaller number of nonredundant genes, our study identified more antibiotic resistance genes than those available in the RGC. Tetracycline, aminoglycoside, and multidrug resistance genes accounted for nearly 70% of the relative abundance in the current catalogue. Slightly higher sharing ratios were shown between the industrialized feedlot pig gut microbiomes and human gut microbiomes than that between the RGC and human counterpart (14.7% versus 12.6% in genes and 94.1% versus 87.7% in functional groups, respectively). Furthermore, a remarkably high number of the antibiotic resistance proteins (*n* =141) were identified to be shared by the pig, human, and mouse resistome, indicating the potential for horizontal transfer of resistance genes. Of the antibiotic resistance proteins shared by pigs and humans, 50 proteins were related to tetracycline resistance, and 49 were related to aminoglycoside resistance.

**IMPORTANCE** The gut microbiota is believed to be closely related to many important physical functions in the host. Comprehensive data on mammalian gut metagenomes has facilitated research on host-microbiome interaction mechanisms, but less is known about pig gut microbiome, especially the gut microbiome in industrialized feedlot pigs, compared with human microbiome. On the other hand, pig production, as an important source of food, is believed to exacerbate the antibiotic resistance in humans due to the abuse of antibiotics in pig production in various parts of the world. This study delineates an intricate picture of swine gut microbiome and antibiotic resistome in industrialized feedlots and may provide insight for the pig producing industry.

## Abstract (precis)

The gut microbiota is believed to be closely related to many important physical functions in the host. Comprehensive data on mammalian gut metagenomes has facilitated research on host-microbiome interaction mechanisms, but less is known about pig gut microbiome, especially the gut microbiome in industrialized feedlot pigs, compared with human microbiome. On the other hand, pig production, as an important source of food, is believed to exacerbate the antibiotic resistance in humans due to the abuse of antibiotics in pig production in various parts of the world. This study delineates an intricate picture of swine gut microbiome and antibiotic resistome in industrialized feedlots and may provide insight for the pig producing industry.

## INTRODUCTION

The gut microbiota is closely related to energy metabolism, immune-system development, and other important physical functions (1, 2). In humans, the interaction mechanisms between gut microbiota and host were widely studied (3). The animal microbiota also modulates various aspects of host activities. However, compared with the human counterparts, less is known about the pig gut microbiota, despite several articles primarily utilizing 16S rRNA gene sequencing or metagenomic sequencing (4–10). There is a paucity of data examining the gut microbiome in industrialized feedlot pigs, which sets back the process to accurately define and explore the specific gene structures and functional profiles. In 2016, as an attractive model, a reference catalogue of swine gut microbiome was established on the basis of metagenome sequencing of 287 pig fecal samples collected from France (100 pigs), Denmark (100 pigs), and China (87 pigs) (8). However, the pigs from China used in Xiao’s study (8) were raised on laboratory farms, which might not fully reflect the swine gut microbiome under field conditions. Pigs on large-scale commercial pig production farms face more complicated environmental factors, such as high population density and heavy antibiotic use (9, 11, 12). A recent study had quantified and characterized the acquired resistance gene pools (resistomes) of 181 pig and 178 poultry farms from nine European countries, showing that the pig and poultry resistomes were very different in abundance and composition and that the total acquired antibiotic gene level was associated with the overall country-specific antimicrobial usage in livestock (9). To characterize the diversity and richness and explore the function and structure of swine gut microbiome in common pig-farming feedlots in China, we sampled feces of pigs from four different industrialized feedlots located in four distant provinces across China and metagenome sequenced using whole-metagenome shotgun (WMS) method.

Antibiotics are the most cost-effective way to treat disease and preserve and improve animal health and therefore are widely used in food animal production. It is noteworthy that the use of antibiotics is particularly frequent on large-scale industrial pig farms in China (13). At the same time, approximately 58% of the veterinary antibiotics consumed are excreted into the environment (12). The emergence of multiple-antibiotic-resistant bacteria has aroused great concern over the abuse of antibiotics. A principal concern is whether these antibiotic-resistant bacteria will be transferred from veterinary animals to human beings. Growing evidence during the past 40 years revealed the link between animal antibiotic use and spread and increase of antibiotic resistance (AR) genes in pathogens (14, 15). However, to date, most methods of monitoring AR genes have been based on quantitative PCR, and the throughput was still limited. The volume of the pig farm industry in China provides us a good opportunity to assess a novel method, metagenome sequencing, to monitor the antibiotic resistance gene diversity and abundance quickly and extensively. Thus, in this study, we investigated the pig gut resistome in four feedlots in China. This will provide a useful reference for future studies.

## RESULTS

### Establishment and assessment of the pig gut gene catalogue in industrialized feedlots.

To characterize the microbial community of the pig gut microbiome in industrialized feedlots in China, first, pig fecal specimens were collected from four feedlots located in four distant provinces across China (see Fig. S1 in the supplemental material). For each feedlot, specimens collected from three to five sampling pigs were mixed uniformly to delineate an overall picture of pig gut microbial community. Microbial DNAs were then extracted and whole-metagenome shotgun sequenced using the Illumina HiSeq3000 platform, which yielded 85.0 Gbp of high-quality data (an average of 21.2 Gbp per feedlot). After *de novo* assembly and gene predicting of the data, we obtained a nonredundant protein-coding gene catalogue containing 3,571,197 genes with an average length of 663 bp. The statistics for these procedures are given in Table S1 in the supplemental material).

10.1128/mSystems.00206-19.1 FIG S1 Geographical distribution of four commercial pig feedlots in this study. Download FIG S1, TIF file, 2.1 MB. Copyright © 2019 Wang et al. 2019 Wang et al This is an open-access article distributed under the terms of the Creative Commons Attribution 4.0 International license.

10.1128/mSystems.00206-19.3 TABLE S1 Sequencing data production, assembly, and gene prediction statistics for each sample. Download Table S1, DOCX file, 0.02 MB. Copyright © 2019 Wang et al. 2019 Wang et al This is an open-access article distributed under the terms of the Creative Commons Attribution 4.0 International license.

**FIG 1:**
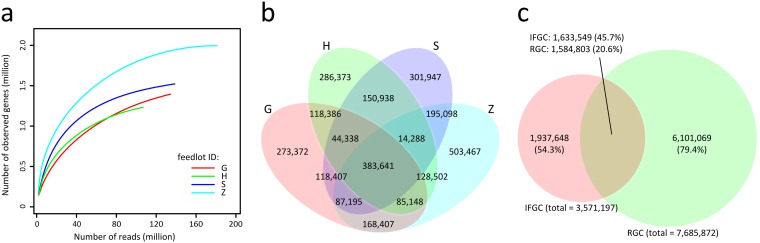
Characterization of pig gut gene catalogue in industrialized feedlots. (a) Rarefaction analysis of pig gut microbiome on each feedlot. The number of observed genes in different feedlots was calculated based on a randomly selected specific numbers of reads (per million) with 20 replacements, and the median was plotted. (b) Gene sharing relationship of four feedlots. (c) Comparison of genes between the IFGC and RGC catalogues, with 54.3% unique nonredundant genes in the IFGC that were not overlapped by RGC.

To evaluate the coverage of the nonredundant gene catalogue, we performed a rarefaction analysis on each feedlot and found that the curve of gene occurrence had nearly plateaued along with the extent of sequencing data (Fig. 1a). Estimation using the incidence-based coverage estimator (ICE) suggested that the gene catalogue covered 87.1% of the gene contents in all samples. Although the feedlots are located in nonneighboring regions of China, we observed that a large proportion (an average of 87.1%) of genes in each feedlot were also found in other feedlots, and 383,641 (10.7%) genes in the nonredundant gene catalogue were shared by all feedlots (Fig. 1b), suggesting that a large overlap of pig gut microbiome existed in the samples. Statistically, adding a new feedlot with the same amount of sequencing data (20 Gbp) would contribute only a low number of new genes (<9.7%) to the current gene catalogue. Thus, our gene catalogue was relatively complete to cover the pig gut genes in the industrialized feedlots in China.

The current pig gut gene catalogue (industrialized feedlot gene catalogue [IFGC]) was compared with the largest reference gene catalogue (RGC) (8), which was constructed based on 287 pig fecal samples collected from France (100 pigs), Denmark (100 pigs), and China (87 pigs). A total of 1,937,648 (54.3%) genes were newly found in the current catalogue (Fig. 1c). This may be due to the fact that all Chinese samples in the RGC were collected from a single laboratorial feedlot in the southern end of China (BGI Ark) (8). Thus, a comprehensive pig gut gene catalogue could be generated by merging the two catalogues (representing 9,672,266 genes).

### Phylogenetic and functional composition of the pig gut microbiome in industrialized feedlots.

**FIG 2:**
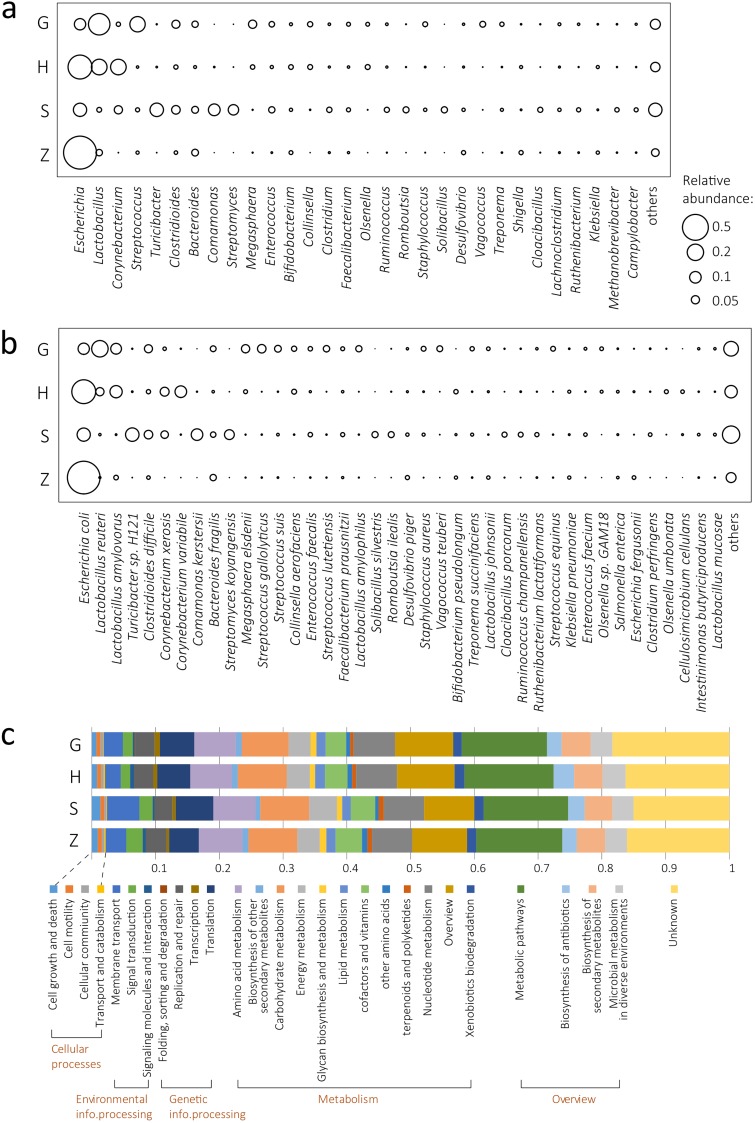
Phylogenetic and functional composition of the pig gut microbiome in industrialized feedlots. (a) Relative abundance of the pig gut microbiome at the genus level in each feedlot. (b) Relative abundance of the pig gut microbiome at the species level in each feedlot. (c) Functional composition of the pig gut microbiome, with functional pathways annotated via the KEGG pathway databases at level B.

The overall phylum-level composition of the pig gut microbiome was similar to that of other mammalians’ gut microbiome, with *Proteobacteria*, *Firmicutes*, and *Actinobacteria* constituting nearly 95% of the gut microbiota (Fig. S2), which was consistent with previous results based on 16S rRNA gene surveys. At the genus level, the genera *Escherichia*, *Bacteroides*, *Comamonas*, *Streptomyces*, and a variety of genera belonging to *Firmicutes* (*Lactobacillus*, *Streptococcus*, * Turicibacter*, *Clostridioides*, etc.) represented the most dominant microbial community (Fig. 2a). The top 27 genera (average relative abundance >0.5%) constituted 90.7% of the relative abundance. At the species level, the pig gut microbial community exhibited a remarkably high level of diversity, with 14 species having an average relative abundance of >1%, 88 species having an average relative abundance of >0.1%, and 235 species having an average relative abundance of >0.01% (Fig. 2b). Escherichia coli were the most abundant bacteria in all samples. The methane-producing archaeon Methanobrevibacter smithii was the most abundant nonbacterial microbe in the pig gut microbiome, accounting for 0.19% of relative abundance in samples.

10.1128/mSystems.00206-19.2 FIG S2 Relative abundance of gut microbiome at phylum level in each feedlot. Download FIG S2, TIF file, 1.3 MB. Copyright © 2019 Wang et al. 2019 Wang et al This is an open-access article distributed under the terms of the Creative Commons Attribution 4.0 International license.

Despite the diversity of composition at the genus and species level, annotating the gene catalogue using the Kyoto Encyclopedia of Genes and Genomes (KEGG) databases revealed a much larger overlap between microbial functional groups from the four feedlots, with the Metabolism pathway showing the highest richness (Fig. 2c).

### Genomes of the dominant species.

To study the genomic features of the dominant microbial species in industrialized feedlots, we *de novo* assembled the reads of the dominant species on each feedlot (see Materials and Methods for detail) and obtained 16 high-completeness draft genomes from the metagenomic data of the analyzed feedlots (Table S2). Notably, seven of these species, including two *Escherichia coli* strains, two *Lactobacillus amylovorus* strains, one *Streptococcus gallolyticus* strain, one *Comamonas kerstersii* strain, and one *Clostridioides difficile* strain, were derived from the human body sites. We further compared these draft genomes with the available reference genomes in the National Center for Biotechnology Information (NCBI) database, to investigate whether the microbial genomes of the pig gut are unique compared with that of other habitats. This procedure revealed that the pig gut bacterial genomes were highly homologous to the genomes isolated from other resources, including human gut, mammal-associated environments, and natural environments. For example, a *Megasphaera elsdenii* genome from feedlot G showed >98% overlap and >99.9% average nucleotide identity (ANI) with a strain known as an animal commensal (*M. elsdenii* 14-14). These taken together indicated the potential frequent exchange of microbiome between the pig gut and other environments.

10.1128/mSystems.00206-19.4 TABLE S2 Detailed information of the draft genomes reconstructed from metagenomic data. Download Table S2, DOCX file, 0.02 MB. Copyright © 2019 Wang et al. 2019 Wang et al This is an open-access article distributed under the terms of the Creative Commons Attribution 4.0 International license.

### Pig gut antibiotic resistome.

**FIG 3:**
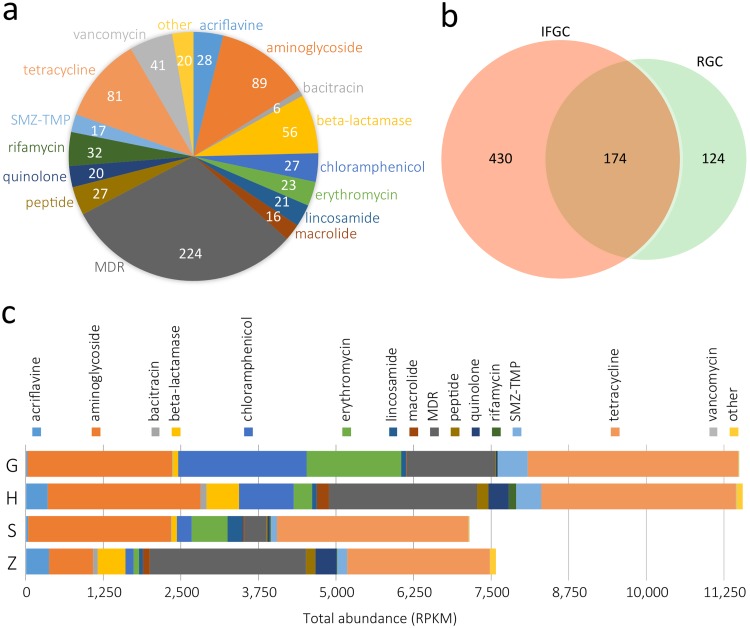
Pig gut antibiotic resistome analysis. (a) Composition of AR genes in the pig gut microbiome in industrialized feedlots. (b) Comparison of AR genes between the IFGC and RGC catalogues. (c) Relative abundance of antibiotic resistance genes in each feedlot.

To explore the pig gut resistome, first, a total of 728 genes in the merged pig gut gene catalogue were identified as antibiotic resistance protein-coding genes based on annotations in the available antibiotic resistance databases (see Materials and Methods and Table S3). Of these proteins, 234 (32.1%) were novel (<90% amino acid similarity to any protein in the NCBI-NR database). Most of the antibiotic resistance (AR) genes were related to multidrug resistance (MDR) (30.8%), aminoglycoside resistance (12.2%), and tetracycline resistance (11.1%) (Fig. 3a). The remaining genes were involved in resistance against various types of antibiotics such as beta-lactamase (7.7%), vancomycin (5.6%), and rifamycin (4.4%). Though the industrialized feedlot gene catalogue contained smaller number of genes than RGC did, it yielded more AR genes in quantity (Fig. 3b), which was also proportionally larger than expected (Pearson’s chi-squared test, *P* = 4 × 10^−114^).

10.1128/mSystems.00206-19.5 TABLE S3 Detailed information of the antibiotic resistance genes identified from pig gut microbiomes. Download Table S3, XLSX file, 0.05 MB. Copyright © 2019 Wang et al. 2019 Wang et al This is an open-access article distributed under the terms of the Creative Commons Attribution 4.0 International license.

We then quantiﬁed the relative abundances of antibiotic resistance genes in pig feedlots. Of all the detected AR genes in the pig gut resistome, tetracycline, aminoglycoside, and multidrug resistance genes played a major role, accounting for nearly 70% of abundance (Fig. 3c). Notably, there were huge differences in diversity and richness of AR genes among the four feedlots, with feedlots G and H topping the list. For example, feedlots G, H, and Z possessed much higher levels of sulphamethoxazole-trimethoprim (SMZ-TMP) resistance genes than feedlot S did, which was mainly due to the wide use of sulfonamides in these three feedlots. In addition, ciprofloxacin-resistant genes *qnrB*, *qnrS1*, and *qnrS2* were detected only in feedlot H. This phenomenon was most likely to be related to antibiotic abuse, since feedlot H used larger amount of antibiotics than the other three feedlots, and ciprofloxacin was only used in feedlot H as a feed additive.

### Comparison of the pig, human, and mouse gut microbiomes and resistomes.

**FIG 4:**
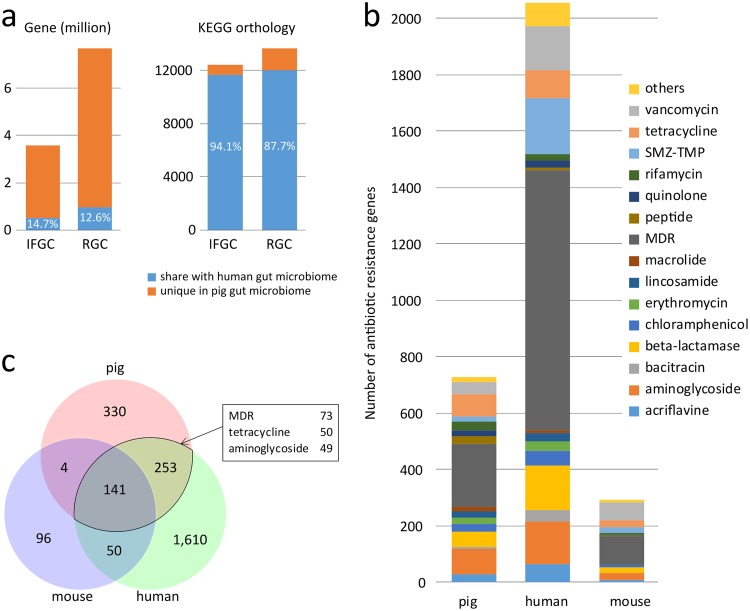
Comparison of the gut antibiotic resistome of pig, human, and mouse. (a) Two nonredundant gene catalogues, the IFGC and RGC, were compared with the integrated human gut gene catalogue (16), and their overlapping rates in genes and functional groups were shown. (b) Comparison of AR types and number of AR genes in the pig, human, and mouse gut resistomes. (c) Pairwise overlap of gut resistomes in pig, human, and mouse.

A previous study revealed that pig gut microbiomes manifested a closer relationship with human gut microbiomes than with mouse gut microbiomes, supporting the potential use of pigs for biomedical research (8). In our data set, similarly, we aligned the industrialized feedlot gene catalogue to the integrated human gut gene catalogue (16) and found that 14.7% of genes and 94.1% of functional groups in the pig gut microbiome were shared by the human counterpart (Fig. 4a). Remarkably, the sharing ratios between the industrialized feedlot pig and human gut microbiomes were slightly higher than that between the RGC and human counterpart (12.6% of genes and 87.7% of functional groups in RGC were shared with the human counterpart, Pearson’s chi-squared test, *P* < 0.001 for both comparisons), suggesting a more frequent gene exchange between livestock field production and human beings.

A total of 2,054 and 291 antibiotic resistance proteins were identified from the human (16) and mouse (17) gut gene catalogues, respectively. The pig gut resistome comprised more diverse and larger amounts of AR proteins than the mouse resistome (184 samples), but fewer AR proteins compared with the human resistome (>1,200 samples) (Fig. 4b). Compared with the resistomes of humans and mice, the pig gut counterpart contained more AR proteins involving tetracycline resistance (*P* = 1 × 10^−8^) and fewer proteins involving vancomycin resistance (*P* = 0.002), probably due to the frequent supplementation of tetracycline in farms in China. Based on a threshold of 95% nucleic acid similarity, a remarkably high percentage of the AR proteins (*n* = 141) were identified to be shared by the pig, human, and mouse resistomes (Fig. 4c). The pairwise overlap was also modest for pig versus human (394 proteins) resistomes and for mouse versus human (191 proteins) resistomes. Of the AR proteins shared by pigs and humans, 50 proteins were related to tetracycline resistance and 49 were related to aminoglycoside resistance, suggesting that these antibiotics were most frequently exchanged between pigs and humans.

## DISCUSSION

The structural and functional composition of the pigs’ gut microbiome could be shaped by external factors (18, 19). Due to its important role in energy utilization and health maintenance in domestic animals, modern industrial farming pays increasing attention to the gut microbiome (20, 21). Otherwise, the microbiota of pig, a close economic animal with human beings, could influence the microbial composition of human beings and environments (22).

Compared with previous studies, our study focused on the comprehensive analysis of gut microbiome and resistome of pigs in Chinese industrialized feedlots by employing metagenome sequencing. In 2016, a reference catalogue of the pig gut microbiome was established by metagenome sequencing of fecal samples from 287 pigs, which were raised in laboratory conditions (8). Our samples, however, came from pigs on industrialized feedlots located in four nonneighboring provinces across China (see Fig. S1 in the supplemental material). Surprisingly, more than half (54.3%) of the genes were newly discovered in the IFRC, suggesting a huge difference in microbiomes between laboratory-raised pigs and farm-raised pigs, which we speculate can be explained by circumstances, such as in-feed antibiotic (4), daily diet (23), growth traits (7), and so on.

By *de novo* assembling reads from dominant species, we obtained 16 high-completeness draft genomes, which share high identities with species from other habitats such as human body sites, mouse, poultry, and even fish. This implies frequent communication between pig microbiota and that of other origins and highlights the need for further study on the communication mechanism due to increasing appeals and policies to encourage the application of pig manure as fertilizers to reduce environmental pollution and to promote energy recycling. In fact, several concerns have been raised in reports stating that improper and unnormalized usage of pig manure as fertilizers increases the AR genes in the soil, perhaps even for a long time (15).

This study documents the breadth and extent of the antibiotic resistance reservoir in large-scale industrial pig feedlots. A previous study using a high-capacity quantitative PCR method to analyze pig feces in large-scale farms, detected 149 resistance genes, of which 63 were enriched in the manure (13). We explored the potential use of metagenomics approach, a novel technology used in the farming industry, in characterizing the antibiotic resistance gene (ARG) profiles of the typical industrial feedlots in China. The major classes of ARGs existing ubiquitously in pig farms included tetracycline, aminoglycoside, and multidrug resistance genes. The gut microbiota of industrialized feedlot pigs retained a high abundance of tetracycline and aminoglycoside genes, which was consistent with the core antibiotic resistome of laboratory pigs (4). However, we obtained more ARGs than the previous studies (8).

We also observed remarkable difference in resistome between IFRC and RGC. Our study produces more data on AR genes despite a smaller number of total gene yields compared with RGC. Remarkably, 234 (32.1%) of these proteins were new (<90% amino acid similarity to any protein in the NCBI-NR database), which expands our knowledge of antibiotic resistance genes. This might be due to the abuse of antibiotics in the domestic pig production industry. It has been reported that the total usage of 36 commonly used antibiotics for pigs in China had reached 48,400 tons in 2013, which accounts for 52.2% of total antibiotic usage (humans [15.6%], chickens [19.6%], and other animals [12.5%]) (24).

MDR genes are dominant in the resistome, accounting for 30.8% of total AR genes, followed by aminoglycoside resistance genes (12.2%), and tetracycline resistance genes (11.1%). Interestingly, the relative abundance of MDR genes is also consistent with antibiotic usage in each feedlot. This is easy to understand because antibiotic abuse puts selection pressure on microbiota resistance. The relationship between antibiotic use and AR genes can also be highlighted by the two findings that ciprofloxacin-resistant genes *qnrB*, *qnrS1*, and *qnrS2* were detected only in feedlot G since ciprofloxacin was employed only in feedlot G as a feed additive and that feedlots G, H, and Z exhibited much higher levels of SMZ-TMP resistance genes than feedlot S did, which is possibly due to heavy usage of SMZ-TMP in these three feedlots (Fig. S1). However, there are several antibiotic resistance genes that emerge from the analysis but act on different antibiotics not given to the pigs (Fig. 3a and c). This might be related to several factors as follows: first, bacteria from the surrounding environment or other animals harbor these genes; second, some AR genes are intrinsic to certain bacteria (25); third, some AR bacteria generated in one feedlot previously may still persist in the location. A core set of 141 AR proteins were shared by the mouse gut gene catalogue (MGC), human gut gene catalogue (HGC), and IFGC. Compared with RGC, more antibiotic resistance genes were shared between IFGC and HGC, indicating that AR genes are actually more frequently exchanged between pigs and humans than expected. Fifty proteins related to tetracycline resistance and 49 proteins related to aminoglycoside resistance are shared by IFGC and HGC, suggesting that these antibiotics were most frequently exchanged between pigs and humans. These results are consistent with previous reports (9, 12, 13).

In conclusion, our study provides a comprehensive analysis of the gut microbiome and resistome of pigs raised in industrialized feedlots in four distant provinces across China. We identified 1,937,648 new genes (54.3%) and 234 novel AR proteins (32.1%) compared with previous RGC data based on samples from laboratory-raised pigs, which indicates a huge difference in the gut microbiome and resistome between lab-raised pigs and commonly reared pigs. The AR proteins shared by IFGC and HGC and the positive correlation between antibiotic usage and AR gene abundances calls for the restricted use of antibiotics in China.

## MATERIALS AND METHODS

### Fecal sampling and DNA extraction.

Pig feces from four distant (a separation of more than 3,500 km) industrialized feedlots that are representative large-scale swine farms located in (from south to north) Guangdong province, Sichuan province, Hebei province, and Heilongjiang province, respectively, in China. The fecal samples were obtained from representative large-scale swine farms with an animal intensity of 10,000 market hogs or more per year. A low-temperature box with ice was used to transport samples from the field to the laboratory; the samples were then immediately frozen and stored at −80°C for further analysis. Three to five fecal samples from each site were mixed, and a total of four mixed samples were prepared for each site. Fecal DNA was extracted using Qiagen DNA extraction kit (Qiagen, Germany) according to the manufacturer’s protocols. The DNA concentration and purity were quantified with TBS-380 and NanoDrop2000, respectively. DNA quality was examined with a 1% agarose gel electrophoresis system.

### Whole-metagenome shotgun sequencing (WMS).

Metagenomic DNA was fragmented to an average size of approximately 300 bp using Covaris M220 (Gene Company Limited, China) for paired-end library construction. Paired-end libraries were prepared by using a TruSeq DNA sample prep kit (Illumina, San Diego, CA, USA). Adapters containing the full complement of sequencing primer hybridization sites were ligated to blunt-end fragments. Paired-end sequencing was performed on Illumina HiSeq3000 platform. High-quality reads were extracted from the raw Illumina sequenced data by trimming the low-quality (Q < 30) bases on the end of reads and filtering “N”-containing, adapter contamination or short-length (<100-bp) reads. After these two steps, high-quality reads with >85% similarity to the pig genomic DNA (Sus scrofa, downloaded from NCBI) were removed.

### Nonredundant gene catalogue construction and annotation.

A *de novo* gene catalogue was constructed based on the WMS sequencing data from the pig fecal samples. High-quality reads were used for *de novo* assembly via MEGAHIT (26), which generated the initial assembly results based on different k-mer sizes (k = 21, 33, 55, 77). *Ab initio* gene identification was performed for all assembled scaffolds using MetaGeneMark (27). Predicted genes were clustered at the nucleotide level by CD-HIT (version v4.5.4) (28), and genes sharing greater than 90% overlap and greater than 95% identity were treated as redundancies.

Taxonomic assignment of the genes was generated by BLASTN against the NCBI-NT database. When BLASTN alignments exceeded 70% best-hit coverage, genes with >90% and 80% sequence identity were used for species- and genus-level taxonomical annotation, respectively. The putative amino acid sequences translated from the nonredundant gene catalogue were aligned by BLASTP against the Kyoto Encyclopedia of Genes and Genomes (KEGG) database (release 83.1) (29). Each protein was assigned a KEGG orthologue (KO) based on the best-hit gene in the database (E value < 1e−5, query_cov > 0.70) with a minimum similarity of 30%.

### Draft genome reconstruction for dominant species.

We established an approach to reconstruct the draft genomes of the high-abundance species (in this study, >5%) in the metagenomic samples. First, metagenomic reads were mapped to the closest reference genomes using SOAP2 (>95% identity) (30). The mapped reads were independently assembled using Velvet (an algorithm for *de novo* short-read assembly for single microbial genomes) (31) using the different k-mer parameters ranging from 39 to 131 to generate the best assembly results. Then, the raw assembled genome was performed scaffolded by SSPACE (32), and gaps were closed by GapFiller (33). The short contigs were filtered with a minimum length threshold of 200 bp. The average nucleotide identity (ANI) between genomes was calculated using the ANIb algorithm which uses BLAST as the underlying alignment method (34). Genomic annotation was implemented using the Prokka pipeline (35), which used a suite of prediction tools to identify the coordinates of genomic features within scaffolds.

### Identification of antibiotic resistance genes.

To identify the antibiotic resistance genes from the pig gut microbiome, amino acid sequences of the genes were aligned against the comprehensive antibiotic resistance database (CARD) (downloaded August 2019) (36) using BLASTP (E value < 1e−5) and assigned to an antibiotic resistance gene by the highest-scoring annotated hit with >80% similarity that covered >70% of the length of the query protein.

### Data availability.

The raw sequencing data acquired in this study have been deposited to the European Bioinformatics Institute (EBI) database under accession number PRJEB31742.
