# Supplementary material for: Characterization of the Pig Gut Microbiome and Antibiotic Resistome in Industrialized Feedlots in China
Source: mSystems. 2019 Dec 17;4(6):e00206-19. doi: 10.1128/mSystems.00206-19 (PMC6918024; doi:10.1128/mSystems.00206-19)
Supplement: TABLE S1 [file mSystems.00206-19-st001.docx]

**Table S1| Sequencing data production, assembly and gene prediction statistics for each sample.**

1. Data production

| Sample | Location | No. of raw reads | Total base (Gbp) | No. of high quality reads | Total base (Gbp) |
| --- | --- | --- | --- | --- | --- |
| G | Guangdong | 133,660,252 | 20.05 | 132,220,030 | 19.83 |
| H | Hebei | 109,441,556 | 16.42 | 108,221,134 | 16.19 |
| S | Sichuan | 144,128,370 | 21.62 | 142,438,656 | 21.35 |
| Z | Heilongjiang | 194,103,384 | 29.12 | 184,178,416 | 27.62 |

1. *De novo* assembly

| Sample | No. of contigs | Total length (bp) | N50 length (bp) | Max length (bp) | Min length (bp) | G+C content (%) | Reads usage (%) | | |
| --- | --- | --- | --- | --- | --- | --- | --- | --- | --- |
|  |  |  |  |  |  |  | PE | SE | Total |
| G | 669,597 | 862,145,870 | 1,528 | 131,695 | 500 | 51.9 | 62.8 | 18.4 | 81.2 |
| H | 544,599 | 719,697,320 | 1,601 | 236,695 | 500 | 53.2 | 63.4 | 17.4 | 80.8 |
| S | 695,565 | 960,116,759 | 1,724 | 555,277 | 500 | 49.7 | 64.6 | 17.6 | 82.2 |
| Z | 1,014,616 | 1,229,232,010 | 1,590 | 297,883 | 500 | 51.3 | 59.3 | 19.6 | 78.9 |

1. Gene prediction

| Sample | No. of genes | Total length (bp) | Average length (bp) | % reads mapped to pig gut gene catalogue | |
| --- | --- | --- | --- | --- | --- |
|  |  |  |  | RGC (7.7M) | Current (3.5M) |
| G | 1,206,879 | 766,245,372 | 634.9 | 49.1 | 60.2 |
| H | 995,517 | 638,308,185 | 641.2 | 43.8 | 61.8 |
| S | 1,350,193 | 841,735,422 | 623.4 | 44.8 | 69.7 |
| Z | 1,727,905 | 1108241973 | 641.4 | 56.7 | 66.1 |

RGC: the reference gene catalogue of pig microbiome constructed from 287 pigs (Xiao *et al*, 2016).
