# Supplementary material for: Characterization of the Pig Gut Microbiome and Antibiotic Resistome in Industrialized Feedlots in China
Source: mSystems. 2019 Dec 17;4(6):e00206-19. doi: 10.1128/mSystems.00206-19 (PMC6918024; doi:10.1128/mSystems.00206-19)
Supplement: TABLE S2 [file mSystems.00206-19-st002.docx]

**Table S2| Detailed information of the draft genomes reconstructed from metagenomic data.**

| Assembled genomes | Relative abundance (%) | No. of contigs | Total length (bp) | N50 length (bp) | Completeness (%) | Closest species/strain in NCBI | Origin | ANI (%) |
| --- | --- | --- | --- | --- | --- | --- | --- | --- |
| feedlot G |  |  |  |  |  |  |  |  |
| *Lactobacillus reuteri* | 20.2 | 145 | 1,905,015 | 118,246 | 97.1 | *Lactobacillus reuteri* I5007 | swine | 96.70 |
| *Escherichia coli* | 9.5 | 317 | 5,474,176 | 289,117 | 98.9 | *Escherichia coli* CE516 | human | 99.17 |
| *Lactobacillus amylovorus* | 8.6 | 156 | 1,961,592 | 169,234 | 98.1 | *Lactobacillus amylovorus* AF08-3 | human | 96.61 |
| *Streptococcus gallolyticus* | 5.4 | 312 | 2,373,119 | 69,492 | 100 | *subsp. gallolyticus* ATCC 43143 | human | 98.91 |
| *Megasphaera elsdenii* | 5.1 | 103 | 2,467,543 | 50,476 | 99.3 | *Megasphaera elsdenii* 14-14 | animal | 99.94 |
| feedlot H |  |  |  |  |  |  |  |  |
| *Escherichia coli* | 42.4 | 71 | 5,320,029 | 355,318 | 100 | *Escherichia coli* XH140A | human | 99.80 |
| *Lactobacillus amylovorus* | 11.2 | 249 | 1,837,347 | 153,230 | 94.1 | *Lactobacillus amylovorus* AF08-3 | human | 98.45 |
| *Corynebacterium variabile* | 10.1 | 517 | 2,996,054 | 85,460 | 97.7 | *Corynebacterium variabile* Mu292 | cheese | 90.50 |
| *Corynebacterium xerosis* | 6 | 660 | 2,592,372 | 108,991 | 91.4 | *Corynebacterium xerosis* GS 1 | yak | 95.19 |
| *Lactobacillus reuteri* | 5 | 241 | 1,782,350 | 81,478 | 96.3 | *Lactobacillus reuteri* JCM 1081 | poultry | 99.64 |
| feedlot S |  |  |  |  |  |  |  |  |
| *Escherichia coli* | 13.1 | 162 | 4,877,930 | 266,145 | 97.1 | *Escherichia coli* swine1 | swine | 98.91 |
| *Turicibacter sp. H121* | 13.5 | 383 | 2,215,033 | 101,809 | 99.0 | *Turicibacter sp. H121* | mouse | 99.22 |
| *Comamonas kerstersii* | 10 | 461 | 3,436,956 | 110,754 | 96.2 | *Comamonas kerstersii* J29 | human | 97.01 |
| *Streptomyces koyangensis* | 7.2 | 352 | 6,038,515 | 189,400 | 89.8 | *Streptomyces koyangensis* VK-A60T | fish | 93.07 |
| *Clostridioides difficile* | 5.5 | 240 | 3,783,438 | 218,094 | 93.3 | *Clostridioides difficile* CD21062 | human | 99.55 |
| feedlot Z |  |  |  |  |  |  |  |  |
| *Escherichia coli* | 79.6 | 93 | 5,479,907 | 142,098 | 100 | *Escherichia coli* LY80 | industry | 99.83 |
